# Genetic Insights Into Skin Diseases and Depression: Evidence From East Asian Mendelian Randomization Analysis

**DOI:** 10.31083/AP47646

**Published:** 2025-10-24

**Authors:** Shuang Lin, Xu Yao

**Affiliations:** ^1^Department of General Medicine, Tongde Hospital of Zhejiang Province, 310000 Hangzhou, Zhejiang, China; ^2^Department of Acupuncture and Massage, Tongde Hospital of Zhejiang Province, 310000 Hangzhou, Zhejiang, China

**Keywords:** East Asian, Mendelian randomization, skin disease, depression

## Abstract

**Background:**

This Mendelian randomization (MR) study systematically examines the causal links between skin disorders and depression in individuals of East Asian descent.

**Methods:**

MR analysis employed summary-level genome-wide association study (GWAS) data from East Asian populations. Exposures included six skin diseases: atopic dermatitis (AD) (*n* = 168,103), urticaria (*n* = 172,083), vitiligo (*n* = 13,327), systemic lupus erythematosus (SLE) (*n* = 51,009), psoriasis (*n* = 69,688) and acne (*n* = 2062). Depression was assessed using major depressive disorder (MDD) data from the Psychiatric Genomics Consortium (*n* = 194,548). The primary analytical methods were the inverse variance weighting (IVW) and Wald Ratio. Sensitivity analyses were conducted to detect heterogeneity and pleiotropy, incorporating Steiger tests to mitigate reverse causation.

**Results:**

In East Asian ancestries, a significant causal relationship was identified between urticaria and an increased risk of MDD (odds ratio [OR] = 1.220, 95% CI 1.022–1.457, *p* = 0.028). No significant causal link was found between psoriasis and MDD. Both findings are in stark contrast to those from previous MR studies of European ancestries. No significant causal associations were observed between AD, vitiligo, SLE, acne and MDD, consistent with previous MR studies in European populations. Sensitivity analyses revealed no significant evidence of heterogeneity or pleiotropy, supporting the robustness of the causal evidence.

**Conclusions:**

This study identifies a significant positive causal relationship between urticaria and MDD risk and no significant association between psoriasis and MDD in East Asian populations, contrasting with previous European findings. Results for other skin diseases align with previous studies. These findings highlight the need for ancestry-specific research to inform personalized prevention and intervention strategies.

## Main Points

1. Identified a causal link between urticaria and depression in East Asian populations.

2. Found no causal relationships between other skin diseases and depression.

3. Employed advanced Mendelian randomization techniques for robust causal inferences.

4. Highlights the importance of ancestry-specific research for personalized health strategies.

5. Advocates for larger, multi-ethnic datasets to validate and expand the findings globally.

## 1. Introduction

Depression is a common mental health disorder, defined by sustained low mood, diminished interest or enjoyment in activities and pervasive fatigue or aversion to engagement. These symptoms often lead to substantial impairments in daily functioning and disruptions in social relationships [1]. Major depressive disorder (MDD), a severe form of depression, is among the most serious mental illnesses globally, with clinical manifestations including persistent sadness and loss of interest lasting at least two weeks [2]. The World Health Organization estimates that the global annual incidence of MDD is approximately 4.4% [3]. The prevalence of depression has consistently increased, especially with the worldwide spread of the Coronavirus Disease 2019 (COVID-19) pandemic. By 2030, MDD is expected to be one of the leading contributors to global disease burden [4]. The impact of depression is profound, being a major contributor to disability worldwide and closely linked to an elevated risk of suicide [5]. Beyond greatly increased psychological and physical distress to individuals, depression also imposes a substantial socio-economic burden, including increased healthcare costs and productivity losses. Despite significant research advances, the exact etiology and pathogenesis of depression remain largely unknown. Currently, the condition is viewed as a multifactorial ailment, shaped by a combination of genetic, neurobiological, psychological and environmental influences [4]. Timely interventions and an emphasis on research into causes are vital for halting increasing disease numbers and enhancement of the quality of life for the many individuals impacted. Identifying and understanding the factors associated with depression are crucial for the development of effective preventive strategies.

Skin diseases are often comorbid with mental health issues [6]. As the most visible organ of the human body, the skin plays a crucial role in self-perception and social interaction. Study have shown that individuals with chronic urticaria (CU) frequently exhibit symptoms of anxiety and depression, with depression occurring at a notably higher rate than in the general population [7]. Additionally, a systematic review of 41 studies reported that up to 62.3% of individuals with vitiligo exhibit depressive symptoms [8]. Another study revealed that patients with atopic dermatitis (AD) are more prone to depression and psychosomatic symptoms when compared to healthy controls [9]. Evidence also indicates a bidirectional relationship between anxiety and depression in individuals with systemic lupus erythematosus (SLE) [10], who demonstrate a depression incidence twice that of the general population, potentially mediated by inflammatory factors [11]. Furthermore, psoriasis patients are reported to be at a higher risk of mental health issues, with depression affecting up to 20% of this population [12]. However, conflicting findings have been presented by other studies [13, 14, 15, 16], leaving the relationship between skin disease and depression controversial. It is important to note that most observational studies examining the association between skin diseases and depression rely heavily on self-reported questionnaires. Such methods are susceptible to measurement bias and are influenced by the subjective emotions of patients. Furthermore, these studies are predominantly observational and are limited by their inability to effectively remove the impact of confounding factors. Although randomized controlled trials are the primary standard for confirming causal relationships, they often face challenges such as high economic cost and ethical constraints, limiting their application in certain fields.

Mendelian randomization (MR) analysis employs genetic variation as an instrumental variable (IVs) to estimate causal relationships between exposure, such as lifestyle factors or biomarkers and disease. This approach inherently avoids confounding factors common in traditional observational studies, as genetic variations are randomly allocated to individuals at conception [17]. By leveraging this “natural experiment”, MR analysis provides “purer” and more reliable causal inferences, offering a principled basis for understanding complex disease mechanisms and the development of effective prevention measures [18]. Interestingly, existing MR studies on the relationship between skin diseases and depression have primarily focused on European ancestries, acknowledging a significant limitation due to the homogeneity of the data [19, 20, 21, 22, 23]. The frequency of single nucleotide polymorphisms (SNPs) and the effect size of associations between SNPs and traits (exposures or outcomes) can vary markedly across different ancestries, which may lead to applicability issues affecting clinical relevance and the formulation of public health policy. This study aims to fill this gap by investigating the causal relationships between different skin diseases and MDD in East Asian populations, offering specific insights for global prevention and intervention strategies.

## 2. Methods

### 2.1 Study Design

**Fig. 1.**
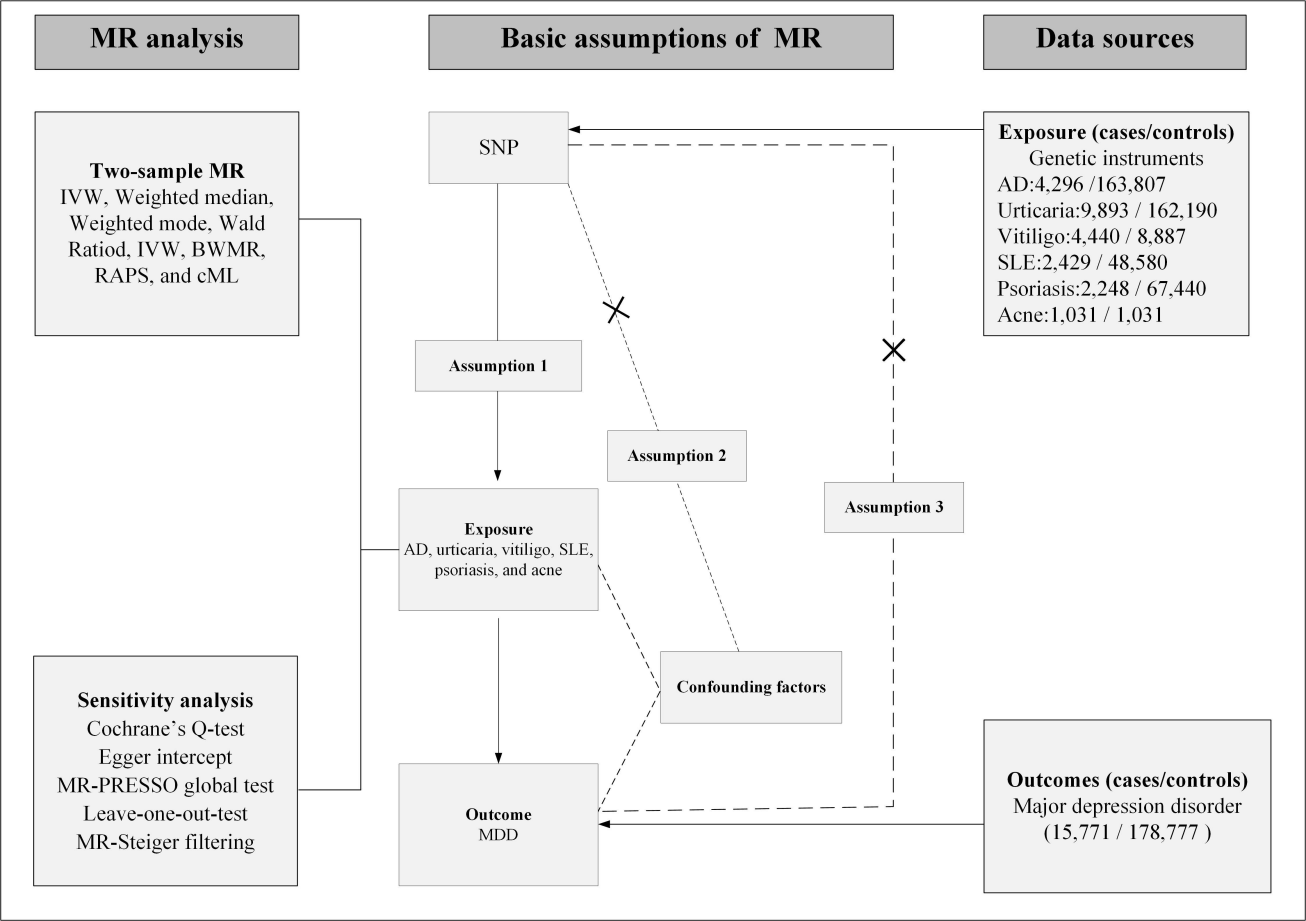
**Study design**. Solid arrows depict the valid causal chain—SNP → Exposure → Outcome—required by MR. Dashed arrows with “×” mark bias-inducing routes that must be absent: (i) SNP → Confounders → Outcome, breaching Assumption 2; (ii) a direct SNP → Outcome path, i.e., horizontal pleiotropy, breaching Assumption 3. The dashed corner brackets the forbidden direct path for clarity. AD, atopic dermatitis; SLE, systemic lupus erythematosus; MDD, major depressive disorder; MR, Mendelian randomization; MR-PRESSO, MR Pleiotropy Residual Sum and Outlier; cML, constrained maximum likelihood; BWMR, Bayesian weighted Mendelian randomization; RAPS, robust adjusted profile score; IVW, inverse-variance weighted; SNP, single nucleotide polymorphism.

Research followed the Strengthening the Reporting of Observational Studies in Epidemiology-Mendelian Randomization (STROBE-MR) standards for the documentation of MR studies [24]. For STROBE-MR, please see the **Supplementary material-STROBE MR checklist fillable**. The choice of SNPs as IVs was directed by the three fundamental principles of MR: (1) The selected genetic variants were associated with the exposure, specifically various skin disease phenotypes. (2) Variants were further evaluated to ensure no association with potential confounding factors. (3) Effects of the genetic variants on the outcome were required to arise exclusively through the exposure, rather than through alternative pathways, to satisfy the core assumptions of MR [25]. Fig. 1 provides a detailed overview of the study design. As a secondary analysis of publicly available data, this study did not require approval from an ethical review committee.

### 2.2 Selection of Genetic Instrumental Variables

(1) Genetic variants were initially selected based on genome-wide significance (*p* < 5 × 10^-8^) and stringent linkage disequilibrium criteria (r^2^ < 0.001 within a 10 MB window). (2) Calculate the F-statistic to identify and exclude weak IVs with an F-statistic below 10, thereby minimizing bias [26]. (3) Further evaluation of the selected genetic variants, was performed with a MR-Steiger test. That analysis was based on the R^2^ values of the genetic variants and the sample size (*n*). Variants that exhibited a stronger association with outcome rather than exposure were excluded to satisfy the third assumption of MR [27, 28]. (4) If relevant SNPs could not be extracted from the outcome dataset, proxy SNPs were not employed to ensure precision (r^2^ > 0.8). (5) SNP alleles linked to both exposure and outcome were standardized, explicitly omitting those that exhibit a moderate effect allele frequency (>0.42) or those that are ambiguous (for instance, G/A and G/C) [29].

### 2.3 Data Sources for Various Skin Diseases and Depression in East Asian Populations

Table 1 (Ref. [30, 31, 32, 33, 34, 35]) gives an extensive overview of the data sources employed. Atopic dermatitis and urticaria were obtained from a cross-ancestry meta-analysis by Sakaue *et al*. [30], which incorporated data from the BioBank Japan project. This study included 220 deeply phenotyped genome-wide association study (GWAS) and integrated meta-analyses with data from the UK Biobank and FinnGen to enhance the resolution of the human genomic atlas. For this research, only East Asian GWAS data were used. AD data consisted of 4296 cases and 163,807 controls, with cases defined by the International Classification of Diseases (ICD)-10-L20, phecode-939, and FinnGen endpoint L12_ATOPIC. Urticaria data included 9893 cases and 162,190 controls, with cases defined by ICD-10-L50, phecode-947, and FinnGen endpoint L12_URTICA_ALLERG.

**Table 1.** **Detailed information of data sources**.

	Ref	Data source	Ancestry	Participants
AD	34594039	Sakaue *et al*. [30]	EAS	4296 cases and 163,807 controls
Urticaria	34594039	Sakaue *et al*. [30]	EAS	9893 cases and 162,190 controls
Vitiligo	38286188	Wang *et al*. [31]	EAS	4440 cases and 8887 controls
SLE	38724181	CMUH. [32]	EAS	2429 cases and 48,580 controls
Psoriasis	38757301	CMUH and BBJ. [33]	EAS	2248 cases and 67,440 controls
Acne	24399259	He *et al*. [34]	EAS	1031 cases and 1031 controls
MDD	34586374	PGC. [35]	EAS	15,771 cases and 178,777 controls

Ref, PMID; CMUH, China Medical University Hospital; BBJ, BioBank Japan; PGC, Psychiatric Genomics Consortium; EAS, East Asianr.

The vitiligo dataset was obtained from Wang *et al*. [31], who combined data from two independent cohorts (4440 cases and 8887 controls). Diagnoses adhered to the Vitiligo European Task Force criteria and were confirmed by at least two dermatologists. Among the 11 identified susceptibility loci, six were located in intronic regions, while five were intergenic. SLE data were derived from the China Medical University Hospital (CMUH) Biobank [32]. This study included 2429 SLE cases and 48,580 controls. SLE was defined using ICD-9-710.0 and ICD-10-M32 and exclusion criteria included individuals without a history of SLE-specific medication use or diagnosed with other autoimmune diseases. An additive genetic model was applied, with logistic regression analysis adjusting for covariates such as sex, age and principal components. The study identified 20 significant genetic loci, including genes such as general transcription factor II-I (*GTF2I*), major histocompatibility complex, class II, DQ beta 1 (*HLA-DQB1*) and signal transducer and activator of transcription 4 (*STAT4*).

The psoriasis GWAS summary statistics were sourced from Yang *et al*. [33], who conducted a meta-analysis combining data from the CMUH Taiwan Biobank and BioBank Japan. This dataset included 2248 psoriasis cases and 67,440 controls. Diagnoses were confirmed by dermatologists using ICD-CM codes L40–L41, as well as ICD-9-CM-696. These SNPs were primarily concentrated on chromosomes 5 and 6. Acne data were also sourced from Yang *et al*. [33], encompassing 1031 cases and 1031 controls of Han Chinese ancestry Severe acne was diagnosed based on grade IV of the Pillsbury system. The study identified two novel susceptibility loci located at 11p11.2 and 1q24.2.

MDD data were sourced from the Psychiatric Genomics Consortium and included in a meta-analysis by Giannakopoulou *et al*. [35], which combined GWAS data from nine cohorts comprising 15,771 MDD cases and 178,777 controls. The identification of MDD was carried out through various methods, such as structured clinical interviews, examination of medical records, questionnaires focused on symptoms and self-report surveys. Results indicated variability in the heritability of MDD across East Asian populations, ranging from 6.5% to 15%.

### 2.4 Statistical Analyses

The main analytical technique employed was the inverse-variance weighted (IVW) method. The choice between fixed-effect and random-effect models was based on the calculated heterogeneity (I^2^). If I^2^ < 50%, the fixed-effect model was deemed more appropriate as it provides more precise estimates of the causal effect’s significance. Conversely, if I^2^ ≥ 50%, the random-effect model was used. For analyses with three or more IVs, Supplementary methods such as the Weighted Median [36] and MR-Egger [37] are employed. Meanwhile, the Weighted Median approach estimates the median causal effect with the assumption that a minimum of 50% of the IVs are valid. Conversely, the MR-Egger method adjusts for potential pleiotropy by regressing Wald ratios, making it particularly useful when all SNPs might be influenced by pleiotropy.

To enhance the robustness and reliability of its primary analytical framework, this MR study employed a diverse range of complementary methods. These techniques were carefully selected to validate the consistency and stability of the findings across different analytical settings. Specifically, the study utilized the debiased inverse-variance weighted (dIVW) approach [38], which addresses potential biases in conventional IVW methods. Additionally, Bayesian weighted Mendelian randomization (BWMR) [39] was applied, leveraging Bayesian principles to account for potential pleiotropy. The robust adjusted profile score (RAPS) [40] method was incorporated to mitigate the impact of outliers and heterogeneity in the instrumental variable estimates. Furthermore, the constrained maximum likelihood (cML) [41] approach was included to ensure robustness under scenarios with complex pleiotropic effects.

Sensitivity analysis began with the use of Cochran’s Q test to assess heterogeneity and calculate the I^2^ statistic [42]. Horizontal pleiotropy was evaluated using MR-Egger regression [43] and the MR Pleiotropy Residual Sum and Outlier (MR-PRESSO) [44] methods, which also identified and excluded outliers [45]. Finally, a leave-one-out analysis was conducted to examine the robustness of the overall causal effect [46]. To address multiple testing, the Bonferroni correction was utilized, setting significant causal evidence at *p* < 0.008 (calculated as 0.05/6), while suggestive causal evidence was defined within the range of 0.008 < *p* < 0.05. Burgess’s online calculator was used to assess the minimum detectable causal effect needed to achieve 80% power [47].

### 2.5 Data Analysis Software and Packages

All statistical analyses were conducted using R software 4.2.2 (R Foundation for Statistical Computing, Vienna, Austria), along with the following packages: TwoSampleMR 0.5.6 (MRC Integrative Epidemiology Unit, University of Bristol, Bristol, UK), MR-PRESSO (version 1.0, Ron Do Laboratory, Icahn School of Medicine at Mount Sinai, New York, NY, USA), MRcML (version 0.9, School of Statistics & Division of Biostatistics, University of Minnesota, Minneapolis, MN, USA), mr.raps (version 0.2, Statistical Laboratory, University of Cambridge, Cambridge, UK), BWMR (version 0.1, Department of Mathematics, The Hong Kong University of Science and Technology, Hong Kong SAR, China), and MendelianRandomization (version 0.9.0, MRC Biostatistics Unit, University of Cambridge, Cambridge, UK).

## 3. Results

### 3.1 Selection and Validation of Genetic Variants

**Fig. 2.**
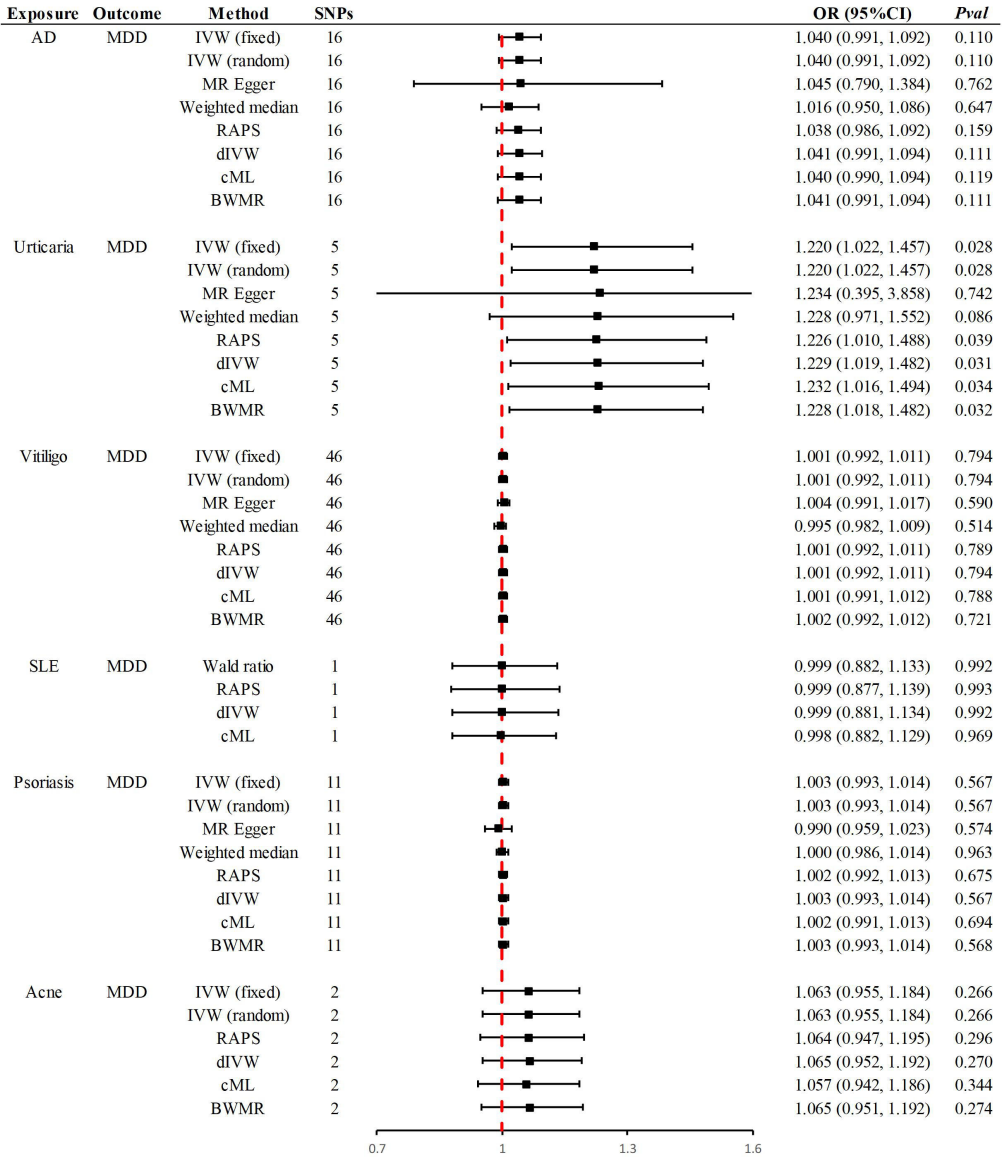
**Summary of Mendelian randomization results**. OR, odds ratio; dIVW, debiased inverse-variance weighted.

The summarized results for the six different skin diseases and their association with MDD are given in Fig. 2. The number of SNPs included in the study ranged from 1 to 46, all of which passed the Steiger test, with the direction confirmed as “TRUE”, effectively filtering out genetic variants that violated MR assumption (3). This ensured the avoidance of reverse causation. All included SNPs had F-statistics greater than 10, minimizing the risk of bias from weak instrument variables. For SLE, the primary analytical method was the Wald Ratio (SNP = 1). For urticaria, no SNPs met the genome-wide significance threshold, necessitating the use of a less stringent threshold to obtain sufficient SNPs for MR analysis (*p* < 5 × 10^-6^). Based on I^2^ statistics, the fixed-effect model was selected for all analyses. The details of the SNPs included in the analysis are given in **Supplementary Table 1**, with scatter plots, funnel plots, forest plots and leave-one-out analyses summarized in **Supplementary Figs. 1–4**.

### 3.2 Impact of Urticaria on MDD Risk

The primary method revealed that a one-standard-deviation increase in genetically predicted urticaria corresponded to a 22% higher risk of MDD [odds ratio (OR) = 1.220, 95% CI 1.022–1.457, *p* = 0.028]. Supplementary methods, including RAPS (OR = 1.226, 95% CI 1.010–1.488, *p* = 0.039), dIVW (OR = 1.229, 95% CI 1.019–1.482, *p* = 0.031), cML (OR = 1.232, 95% CI 1.016–1.494, *p* = 0.034) and BWMR (OR = 1.228, 95% CI 1.018–1.482, *p* = 0.032), provided consistent causal evidence (Fig. 2). At an OR of 1.220, the analysis provided 92% statistical power, further strengthening the causal evidence. A forest plot highlighted rs375132703 as the genetic variant with the most significant causal effect. Leave-one-out analysis verified that this association was not influenced by any single SNP. Furthermore, no evidence of heterogeneity or pleiotropy was detected, reinforcing the robustness of the findings (Table 2). However, the significance level did not exceed the Bonferroni corrected *p*-value, so the relationship between urticaria and MDD is considered a potential causal association.

**Table 2.** **Summary of sensitivity results**.

Exposure	Outcome	MR-Egger regression			MR-PRESSO			Cochrane’s Q-IVW				Effect model	Steiger_test	
		Intercept	SE	*pval*	RSS_obs_	*pval*_global test_	Outlier	*I* ^2^	*Q*	*Q_df*	*Q_pval*		Direction	*pval*
AD	MDD	–0.001	0.027	0.974	11.825	0.777	NA	0	10.482	15	0.788	Fixed	TRUE	9.31 × 10 ^-69^
Urticaria	MDD	–0.001	0.055	0.985	5.503	0.525	NA	0	3.563	4	0.468	Fixed	TRUE	2.81 × 10 ^-11^
Vitiligo	MDD	–0.002	0.004	0.606	40.723	0.753	NA	0	38.147	45	0.755	Fixed	TRUE	0
SLE	MDD	NA	NA	NA	NA	NA	NA	NA	NA	NA	NA	NA	TRUE	1.31 × 10 ^-19^
Psoriasis	MDD	0.016	0.020	0.438	10.995	0.547	NA	0	9.044	10	0.528	Fixed	TRUE	0
Acne	MDD	NA	NA	NA	NA	NA	NA	0	0.756	1	0.385	Fixed	TRUE	1.12 × 10 ^-15^

NA, not available.

### 3.3 Other Skin Diseases and Their Non-Causal Association With MDD

No significant causal associations were identified between other skin diseases and MDD. Specifically, in East Asian populations, the genetic susceptibility to AD, vitiligo, SLE, psoriasis and acne showed no causal relationship with an increased risk of MDD. Supplementary Methods confirmed the consistency of these causal directions. At ORs of 1.040, 1.001, 0.999, 1.003 and 1.063, the statistical power was 65%, 6%, 5%, 14% and 34%, respectively. The leave-one-out examination verified that none of the individual SNPs affected the identified null associations. Furthermore, there was no indication of heterogeneity or horizontal pleiotropy, which reinforces the reliability and strength of the results (Table 2).

## 4. Discussion

This two-sample MR study is the first systematic exploration of the causal relationships between six distinct skin diseases and MDD within East Asian populations. The analysis revealed a significant causal link between genetic susceptibility to urticaria and a heightened risk of MDD. In contrast, no causal relationship was identified between psoriasis and MDD. Notably, these results diverge significantly from prior causal evidence reported in European populations. Furthermore, the study demonstrated no causal associations between the genetic susceptibilities to AD, SLE, vitiligo, acne and MDD risk, which aligns with MR findings from European populations.

A meta-analysis involving 25 cohorts found that nearly one-third of CU patients have at least one underlying mental disorder, including MDD [48]. Interestingly, this study is the first to identify a causal association between urticaria and an increased risk of MDD in East Asian populations, a finding that contrasts sharply with MR studies conducted in European populations [23]. Previous epidemiological studies have highlighted conflicting evidence regarding the association between urticaria and MDD [49, 50]. Furthermore, research by Tzur Bitan* et al*. [51] demonstrated that the psychological impact of urticaria varies based on individual characteristics, age and socioeconomic status, complicating the establishment of a causal relationship. It should be acknowledged that this study applied a less stringent threshold for the urticaria phenotype to include sufficient SNPs for MR analysis. Furthermore, the phenotype was defined as a summary phenotype (ICD-10-L50), whereas previous studies have focused more specifically on the association between CU and depression. This broader definition may account for the differing findings on disease associations. Future studies utilizing larger GWAS datasets are needed to validate the findings reported here.

Previous MR studies have extensively investigated the causal relationship between psoriasis and MDD in European populations, but the results remain inconsistent [23, 52, 53]. In European ancestries, MR studies by Wang *et al*. [53] and Chu *et al*. [52] supported psoriasis as a risk factor for MDD, whereas the findings of Mo *et al*. [23] contradicted this conclusion. Due to the lack of a systematic review on this topic, this study did not further explore the relationship. However, the findings of this study align with those of Mo *et al*. [23], emphasizing the negative association between psoriasis and MDD across both European and East Asian ancestries. In this study, genetic variants were used as IVs, recognizing that significant differences in genetic composition exist across populations of different ancestries. These genetic differences may influence the expression and function of disease susceptibility genes, contributing to the ethnic-specific causal association between psoriasis, urticaria and MDD. Additionally, environmental factors and lifestyle differences between European and East Asian populations must be considered. Psoriasis, urticaria and MDD are likely influenced by complex interactions between genetic and environmental factors, with gene-environment interactions potentially exhibiting distinct patterns in different regions.

Previous observational studies have been influenced by various confounding factors and reverse causation, often resulting in biased findings that reflect correlations rather than causal relationships. This study, using MR analysis, revealed negative causal associations between vitiligo, SLE, AD, acne and MDD in East Asian populations, consistent with MR findings in European populations [19, 20, 21, 22]. First, these skin diseases may primarily develop through pathways unrelated to MDD. For instance, vitiligo and AD are commonly associated with autoimmune responses and skin barrier dysfunction, mechanisms that are unlikely to directly influence the neurobiological processes affecting mental health. Secondly, it is noteworthy that the potential link between depression and skin diseases may partly be mediated through inflammatory pathways. Kouba *et al*. [54], reported significantly elevated serum levels of pro-inflammatory cytokines in patients with depression. These cytokines are implicated not only in the pathophysiology of depression but also in the onset and progression of various skin disorders. For example, increased levels of interleukin (IL)-6 and tumor necrosis factor (TNF)-α, which exacerbate immune system hyperactivity and compromise skin barrier function, often result in chronic inflammation observed in conditions such as urticaria and atopic dermatitis [55, 56]. Liu *et al*.’s [57] MR analysis, systematically investigates the causal relationships between inflammatory cytokines and multiple inflammatory skin diseases, which could provide a biological basis for the causal connection between depression and skin diseases. Finally, the negative findings in this study may be attributed to the limited sample size, which resulted in insufficient statistical power to confirm causal relationships. This limitation aligns with observations from previous MR studies [21]. It underscores the need for larger datasets in future research to validate the associations between other skin diseases and MDD, ensuring the accuracy and reliability of findings.

This study underscores the importance of conducting disease association research in diverse populations and regional contexts. The genetic backgrounds and environmental factors of different ethnic groups and regions may significantly influence the pathophysiological mechanisms of diseases. Research should incorporate larger datasets and cross-population evidence (e.g., South Asian, Hispanic, African populations) to further validate these causal associations and explore their biological mechanisms. Additionally, future studies should consider investigating the potential links between urticaria subtypes and mental health to provide more targeted therapeutic strategies. Finally, it is crucial to note that MR studies rely on the quality and suitability of genetic instrumental variables. Therefore, research should include larger cohort studies and evidence-based approaches combined with MR analysis to achieve triangulation, thereby strengthening the causal conclusions.

This study offers several notable strengths. Foremost, it reports the first MR analysis to comprehensively investigate the causal relationships between diverse skin diseases and MDD specifically within East Asian populations. This focus expands the applicability of the findings to populations beyond those of European ancestry. Second, advanced MR methods, including RAPS, cML, dIVW and BWMR, were utilized, providing multidimensional validation and ensuring robust causal evidence. Additionally, the Steiger test was utilized to address potential reverse causation. Nonetheless, this study is not without limitations. The reliance on publicly available summary-level GWAS data meant that MR analysis could not be performed for skin diseases such as rosacea, alopecia areata and xerosis, as no GWAS data currently exists for these conditions in East Asian populations. Research should focus on generating GWAS datasets from these populations and diseases. Moreover, reliance on summary-level data limited the ability to perform subgroup analyses and hindered further exploration of urticaria subtypes. While GWAS data on urticaria subtypes have been published for European populations, such as in the FinnGen cohort, larger GWAS datasets for urticaria in Asian populations are needed to address these gaps.

## 5. Conclusions

This MR study, utilizing GWAS data from East Asian populations, identified a causal association between urticaria and an elevated risk of MDD. In contrast, no causal link was observed between psoriasis and the risk of MDD. These findings contrast sharply with previous MR studies conducted in European populations. Further, they underscore the importance of personalized mental health assessments and interventions for patients with skin diseases across different ancestries. In clinical practice, early identification and management of depression risk in urticaria patients are particularly critical. Finally, this study emphasizes the necessity of conducting disease association research across diverse populations and regions, providing valuable insights for the development of global healthcare strategies.

## Availability of Data and Materials

Data for MDD are available for download at the Psychiatric Genomics Consortium (https://pgc.unc.edu/for-researchers/download-results/), and data for AD, Urticaria, Vitiligo, SLE, AD, Urticaria, Vitiligo, SLE, Psoriasis, Acne are available in the GWAS Catalog (https://www.ebi.ac.uk/gwas/) based on their PMIDs, or through the original GWAS at the Data Statement.

## Supplementary Material

Supplementary material associated with this article can be found, in the online version, at https://doi.org/10.31083/AP47646.
