# Supplementary material for: Genetic Insights Into Skin Diseases and Depression: Evidence From East Asian Mendelian Randomization Analysis
Source: Alpha Psychiatry. 2025 Oct 24;26(5):47646. doi: 10.31083/AP47646 (PMC12593751; doi:10.31083/AP47646)
Supplement: Supplementary file 1 [file 2757-8038-26-5-47646-s1.zip › Supplementary Figs. 1-4.docx]

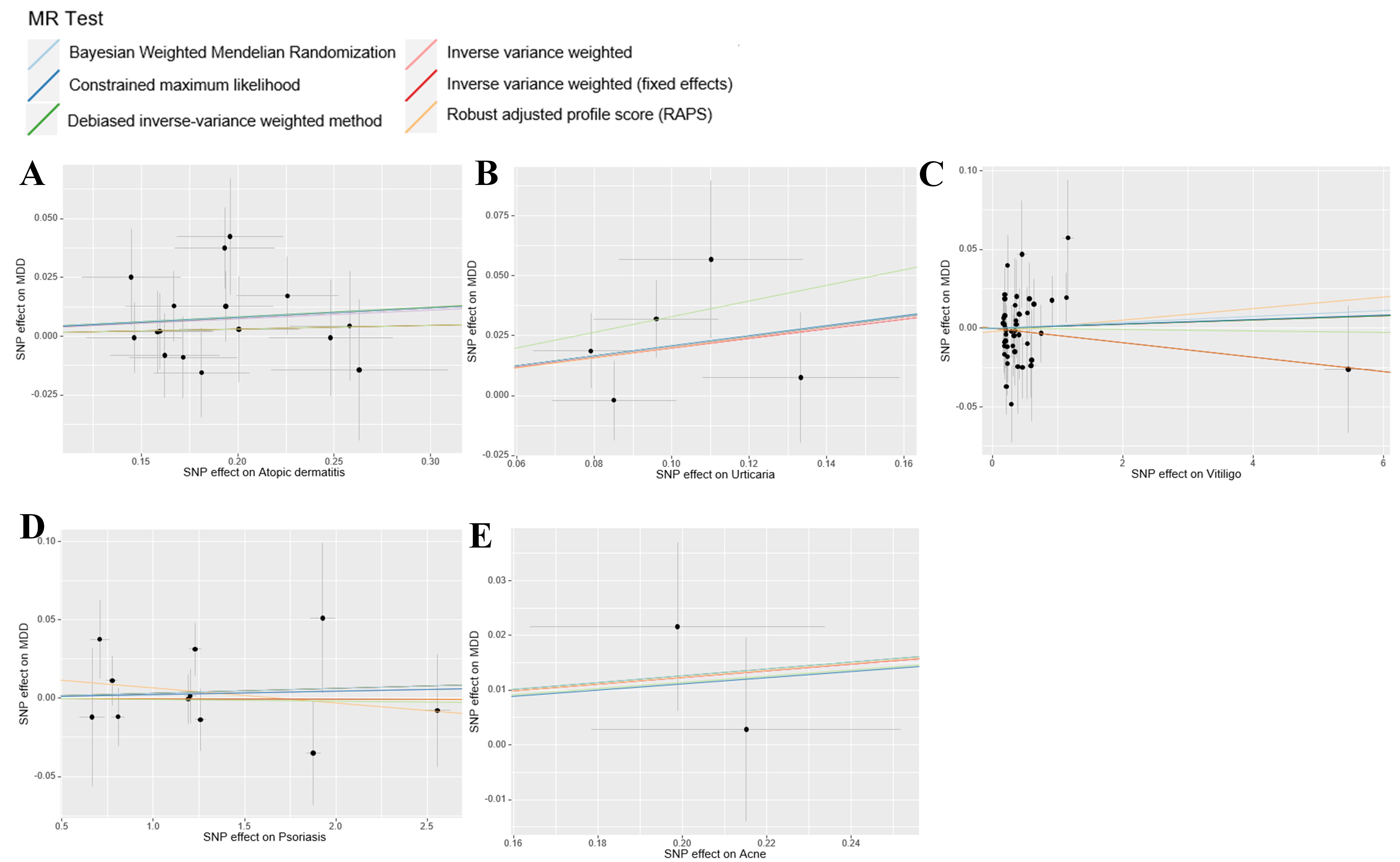


**Supplementary Fig. 1** Genetic associations with skin diseases (horizontal axis, standard deviation units) and with MDD (vertical axis, log odds ratios) at a genome- wide level of significance. (A) AD on MDD (B) Urticaria on MDD (C) Vitiligo on MDD (D) Psoriasis on MDD (E) Acne on MDD.


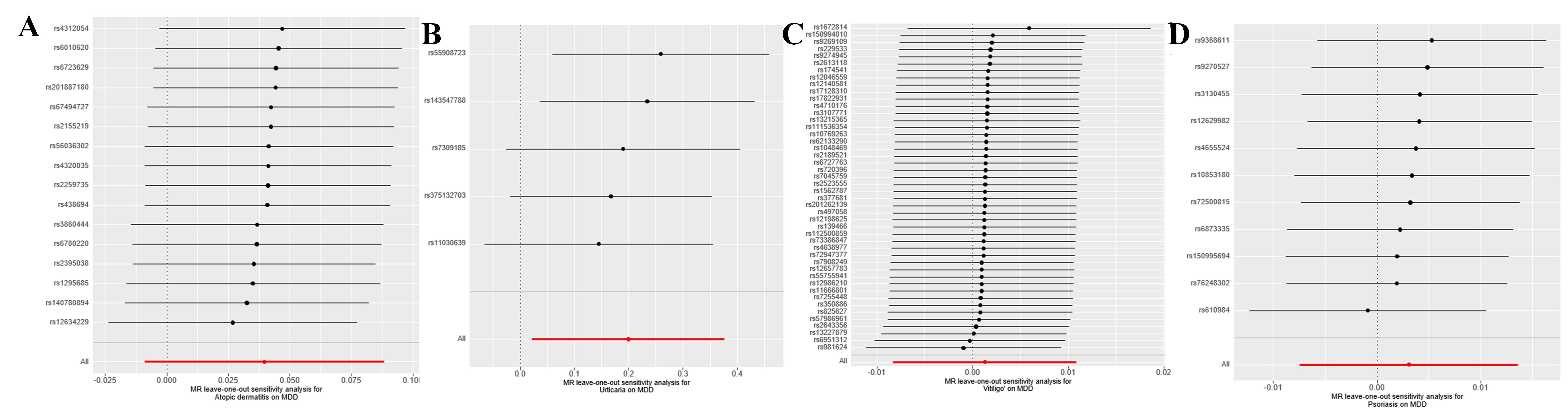


**Supplementary Fig. 2** Leave-one-out plot for MR analysis of skin diseases on MDD. (A) AD on MDD (B) Urticaria on MDD (C) Vitiligo on MDD (D) Psoriasis on MDD


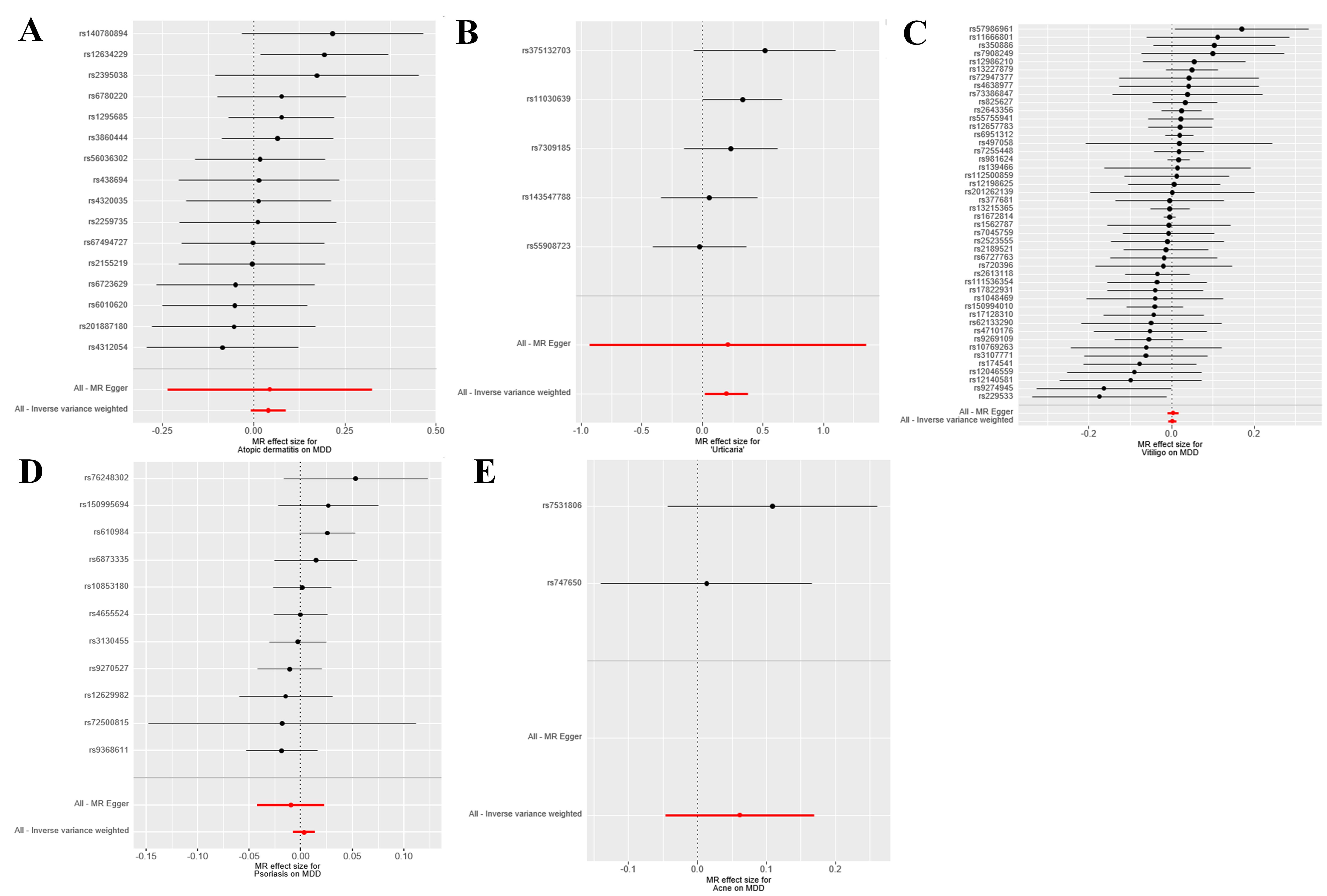


**Supplementary Fig. 3** Single-SNP analysis forest plots of the effect of skin diseases on MDD phenotypes. Point estimates represent the variant-specific ratio estimates for each SNP (in black), and the inverse-variance weighted (IVW) estimate (in red). Horizontal lines represent 95% confidence intervals around the variant-specific ratio estimates and the IVW estimate. (A) AD on MDD (B) Urticaria on MDD (C) Vitiligo on MDD (D) Psoriasis on MDD (E) Acne on MDD.


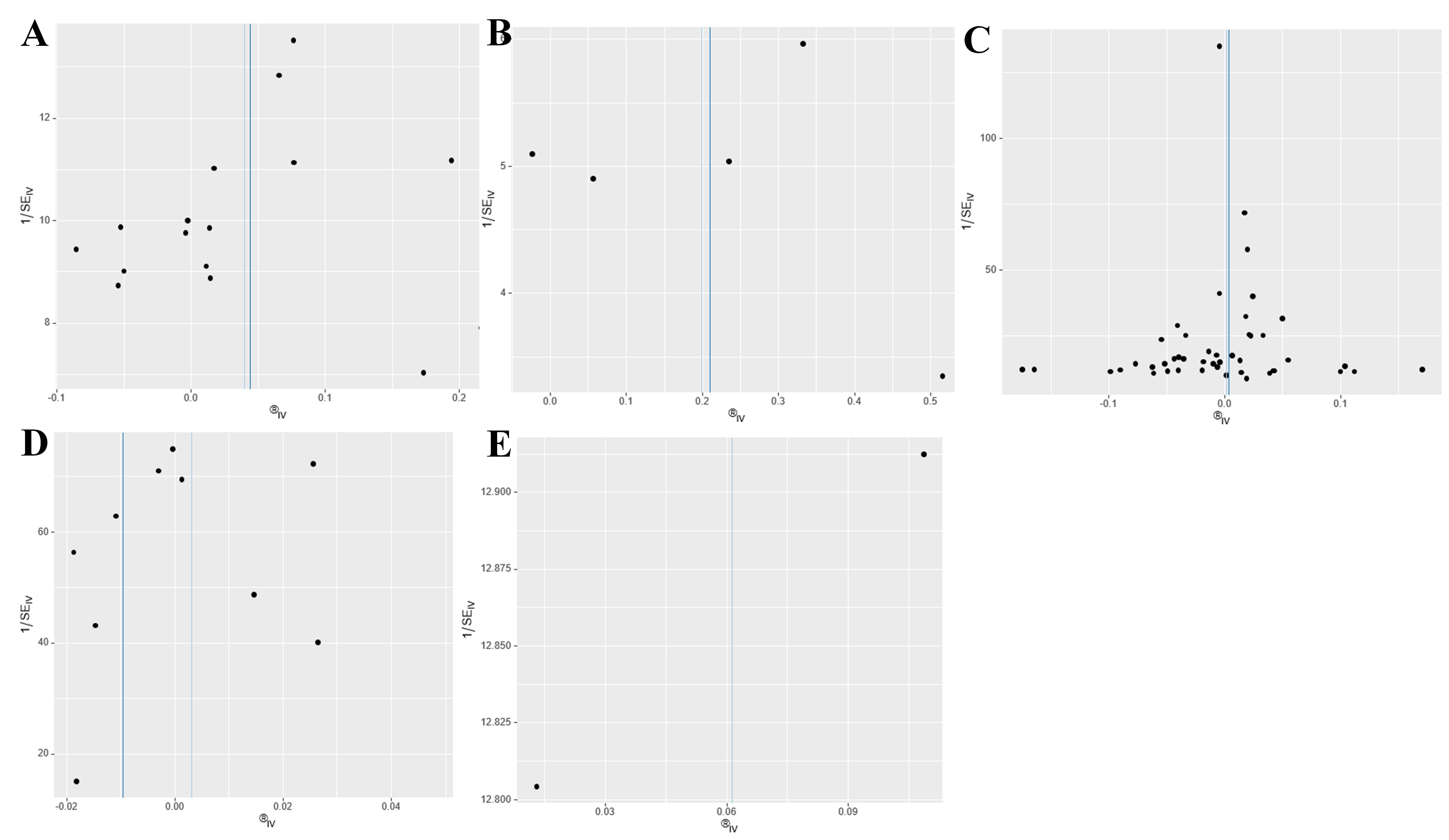


**Supplementary Fig. 4** Funnel plot of instrument precision against instrumental variable estimates for each genetic variant separately for Mendelian randomization analysis of skin diseases on MDD risk. (A) AD on MDD (B) Urticaria on MDD (C) Vitiligo on MDD (D) Psoriasis on MDD (E) Acne on MDD.
