# Supplementary material for: Genetic Insights Into Skin Diseases and Depression: Evidence From East Asian Mendelian Randomization Analysis
Source: Alpha Psychiatry. 2025 Oct 24;26(5):47646. doi: 10.31083/AP47646 (PMC12593751; doi:10.31083/AP47646)
Supplement: Supplementary file 1 [file 2757-8038-26-5-47646-s1.zip › Supplementary material-STROBE MR checklist fillable.docx]

**STROBE-MR checklist of recommended items to address in reports of Mendelian randomization studies**^1^ ^2^

| **Item No.** | **Section** | **Checklist item** | **Page No.** | **Relevant text from manuscript** |
| --- | --- | --- | --- | --- |
| 1 | **TITLE and ABSTRACT** | Indicate Mendelian randomization (MR) as the study’s design in the title and/or the abstract if that is a main purpose of the study | 1,2 | Genetic Insights into Skin Diseases and Depression: Evidence from East Asian Mendelian Randomization Analysis |
|  | **INTRODUCTION** |  |  |  |
| 2 | **Background** | Explain the scientific background and rationale for the reported study. What is the exposure? Is a potential causal relationship between exposure and outcome plausible? Justify why MR is a helpful method to address the study question | 3 | Although Randomized Controlled Trials (RCTs) are the gold standard for confirming causal relationships, they often face challenges such as high economic costs and ethical constraints, limiting their application in certain fields. Mendelian randomization (MR) analysis, which uses genetic variations as instrumental variables (IVs) to estimate the causal relationship between exposures (such as lifestyle factors or biomarkers) and diseases, naturally avoids confounding factors present in traditional observational studies, as these genetic variations are randomly assigned to individuals at conception |
| 3 | **Objectives** | State specific objectives clearly, including pre-specified causal hypotheses (if any). State that MR is a method that, under specific assumptions, intends to estimate causal effects | 3 | This study aims to address this gap by exploring the causal links between various skin diseases and MDD in East Asian populations, providing tailored insights for global prevention and intervention measures. |
|  | **METHODS** |  |  |  |
| 4 | **Study design and data sources** | Present key elements of the study design early in the article. Consider including a table listing sources of data for all phases of the study. For each data source contributing to the analysis, describe the following: | 4 | **Table 1** provides a detailed overview of all data sources utilized in this MR study. |
|  | a) | Setting: Describe the study design and the underlying population, if possible. Describe the setting, locations, and relevant dates, including periods of recruitment, exposure, follow-up, and data collection, when available. | 4 | **Fig. 1** provides a detailed overview of the study design. |
|  | b) | Participants: Give the eligibility criteria, and the sources and methods of selection of participants. Report the sample size, and whether any power or sample size calculations were carried out prior to the main analysis | 4 | **Table 1** provides a detailed overview of all data sources utilized in this MR study. |
|  | c) | Describe measurement, quality control and selection of genetic variants | 4 | **Selection of genetic instrumental variables** |
|  | d) | For each exposure, outcome, and other relevant variables, describe methods of assessment and diagnostic criteria for diseases | 4,5 | 1. Selection of genetic instrumental variables  2. Data Sources for Various Skin Diseases and Depression in East Asian Populations |
|  | e) | Provide details of ethics committee approval and participant informed consent, if relevant | 4 | As this study involves a secondary analysis of publicly available data, it does not require ethical committee approval. Detailed information on the necessary ethical approvals, participant consent, and eligibility criteria for each GWAS can be found in the respective publications. |
| 5 | **Assumptions** | Explicitly state the three core IV assumptions for the main analysis (relevance, independence and exclusion restriction) as well assumptions for any additional or sensitivity analysis | 4 | **Study design** |
| 6 | **Statistical methods: main analysis** | Describe statistical methods and statistics used | 4,5,6 |  |
|  | a) | Describe how quantitative variables were handled in the analyses (i.e., scale, units, model) | 4 | **Selection of genetic instrumental variables** |
|  | b) | Describe how genetic variants were handled in the analyses and, if applicable, how their weights were selected | 4 | **Selection of genetic instrumental variables** |
|  | c) | Describe the MR estimator (e.g. two-stage least squares, Wald ratio) and related statistics. Detail the included covariates and, in case of two-sample MR, whether the same covariate set was used for adjustment in the two samples | 5 | **Statistical Analyses** |
|  | d) | Explain how missing data were addressed | 4 | If relevant SNPs cannot be extracted from the outcome dataset, do not use proxy SNPs to ensure precision (r^2^ > 0.8). |
|  | e) | If applicable, indicate how multiple testing was addressed | 6 | Bonferroni correction was applied, with significant causal evidence defined as *P* < 0.008(0.5/6), and suggestive causal evidence defined as *P* < 0.05 & *P* > 0.008. |
| 7 | **Assessment of assumptions** | Describe any methods or prior knowledge used to assess the assumptions or justify their validity | 4 | **Selection of genetic instrumental variables** |
| 8 | **Sensitivity analyses and additional analyses** | Describe any sensitivity analyses or additional analyses performed (e.g. comparison of effect estimates from different approaches, independent replication, bias analytic techniques, validation of instruments, simulations) | 6 | In the MR analysis, meticulous validation procedures were implemented to maintain the integrity and veracity of the analytical process. |
| 9 | **Software and pre-registration** |  |  |  |
|  | a) | Name statistical software and package(s), including version and settings used | 6 | **Data analysis software and packages** |
|  | b) | State whether the study protocol and details were pre-registered (as well as when and where) | 6 | **Data analysis software and packages** |
|  | **RESULTS** |  |  |  |
| 10 | **Descriptive data** |  |  |  |
|  | a) | Report the numbers of individuals at each stage of included studies and reasons for exclusion. Consider use of a flow diagram | 6 | The summarized results for the six different skin diseases and their association with MDD are presented in **Fig. 2**. |
|  | b) | Report summary statistics for phenotypic exposure(s), outcome(s), and other relevant variables (e.g. means, SDs, proportions) | 6 | The primary method indicated that a one standard deviation (SD) increase in genetically predicted urticaria was associated with a 22% increase in the risk of MDD |
|  | c) | If the data sources include meta-analyses of previous studies, provide the assessments of heterogeneity across these studies | NA |  |
|  | d) | For two-sample MR:  i.  Provide justification of the similarity of the genetic variant-exposure associations between the exposure and outcome samples  ii.  Provide information on the number of individuals who overlap between the exposure and outcome studies | 6 | Results |
| 11 | **Main results** |  |  |  |
|  | a) | Report the associations between genetic variant and exposure, and between genetic variant and outcome, preferably on an interpretable scale | 6 | Details of the SNPs used in the analysis are provided in **Table S1** |
|  | b) | Report MR estimates of the relationship between exposure and outcome, and the measures of uncertainty from the MR analysis, on an interpretable scale, such as odds ratio or relative risk per SD difference | 6 | The primary method indicated that a one standard deviation (SD) increase in genetically predicted urticaria was associated with a 22% increase in the risk of MDD |
|  | c) | If relevant, consider translating estimates of relative risk into absolute risk for a meaningful time period | 6 | The primary method indicated that a one standard deviation (SD) increase in genetically predicted urticaria was associated with a 22% increase in the risk of MDD |
|  | d) | Consider plots to visualize results (e.g. forest plot, scatterplot of associations between genetic variants and outcome versus between genetic variants and exposure) | 6 | scatter plots, funnel plots, forest plots, and leave-one-out analyses are summarized in **Fig. S1–S4**. |
| 12 | **Assessment of assumptions** |  |  |  |
|  | a) | Report the assessment of the validity of the assumptions | 6 | The number of SNPs included in the study ranged from 1 to 46, all of which passed the Steiger test, with the direction confirmed as "TRUE," effectively filtering out genetic variants that violated MR assumption III. |
|  | b) | Report any additional statistics (e.g., assessments of heterogeneity across genetic variants, such as *I^2^*, Q statistic or E-value) | 7 | No evidence of heterogeneity or pleiotropy was observed, supporting the robustness of the results **(Table 2)**. |
| 13 | **Sensitivity analyses and additional analyses** |  |  |  |
|  | a) | Report any sensitivity analyses to assess the robustness of the main results to violations of the assumptions | 7 | No evidence of heterogeneity or pleiotropy was observed, supporting the robustness of the results **(Table 2)**. |
|  | b) | Report results from other sensitivity analyses or additional analyses | 7 | No evidence of heterogeneity or pleiotropy was observed, supporting the robustness of the results **(Table 2)**. |
|  | c) | Report any assessment of direction of causal relationship (e.g., bidirectional MR) | 6 | The number of SNPs included in the study ranged from 1 to 46, all of which passed the Steiger test, with the direction confirmed as "TRUE," effectively filtering out genetic variants that violated MR assumption III. |
|  | d) | When relevant, report and compare with estimates from non-MR analyses | NA |  |
|  | e) | Consider additional plots to visualize results (e.g., leave-one-out analyses) | 6 | scatter plots, funnel plots, forest plots, and leave-one-out analyses are summarized in **Fig. S1–S4**. |
|  | **DISCUSSION** |  |  |  |
| 14 | **Key results** | Summarize key results with reference to study objectives | 7 | This two-sample MR study systematically investigated the causal relationships between six different skin diseases and MDD in East Asian populations for the first time. It identified a causal association between the genetic susceptibility to urticaria and an increased risk of MDD, while confirming no direct causal relationship between psoriasis and MDD. These findings contrast sharply with previous causal evidence reported in European populations. Furthermore, the study demonstrated no causal associations between the genetic susceptibilities to AD, SLE, vitiligo, acne, and MDD risk, which aligns with MR findings from European populations. |
| 15 | **Limitations** | Discuss limitations of the study, taking into account the validity of the IV assumptions, other sources of potential bias, and imprecision. Discuss both direction and magnitude of any potential bias and any efforts to address them | 8 | However, this study also has limitations. The reliance on publicly available summary-level GWAS data meant that MR analysis could not be performed for skin diseases such as rosacea, alopecia areata, and xerosis, as no GWAS data in East Asian populations currently exist for these conditions. Future research should focus on generating GWAS datasets for these diseases in East Asian populations. Moreover, the use of summary-level data precluded subgroup analyses and prevented further investigation into subtypes of urticaria. While GWAS data on urticaria subtypes have been published in European populations, such as in the FinnGen cohort, larger GWAS datasets for urticaria in Asian populations are needed to address these gaps in future studies |
| 16 | **Interpretation** |  |  |  |
|  | a) | Meaning: Give a cautious overall interpretation of results in the context of their limitations and in comparison with other studies | 8,9 | This study revealed distinct causal relationships between East Asian and European ancestries, highlighting a causal association between urticaria and an increased risk of MDD, while confirming no causal relationship between psoriasis and MDD. |
|  | b) | Mechanism: Discuss underlying biological mechanisms that could drive a potential causal relationship between the investigated exposure and the outcome, and whether the gene-environment equivalence assumption is reasonable. Use causal language carefully, clarifying that IV estimates may provide causal effects only under certain assumptions | 7,8 | Discussion of the second and third paragraphs |
|  | c) | Clinical relevance: Discuss whether the results have clinical or public policy relevance, and to what extent they inform effect sizes of possible interventions | 7,8 | Discussion of the second and third paragraphs |
| 17 | **Generalizability** | Discuss the generalizability of the study results (a) to other populations, (b) across other exposure periods/timings, and (c) across other levels of exposure | 8 | Discussion of the last paragraph |
|  | **OTHER INFORMATION** |  |  |  |
| 18 | **Funding** | Describe sources of funding and the role of funders in the present study and, if applicable, sources of funding for the databases and original study or studies on which the present study is based | 9 | Corresponding chapters |
| 19 | **Data and data sharing** | Provide the data used to perform all analyses or report where and how the data can be accessed, and reference these sources in the article. Provide the statistical code needed to reproduce the results in the article, or report whether the code is publicly accessible and if so, where | 9 | Corresponding chapters |
| 20 | **Conflicts of Interest** | All authors should declare all potential conflicts of interest | 9 | Corresponding chapters |

This checklist is copyrighted by the Equator Network under the Creative Commons Attribution 3.0 Unported (CC BY 3.0) license.

1. Skrivankova VW, Richmond RC, Woolf BAR, Yarmolinsky J, Davies NM, Swanson SA, et al. Strengthening the Reporting of Observational Studies in Epidemiology using Mendelian Randomization (STROBE-MR) Statement. JAMA. 2021;under review.

2. Skrivankova VW, Richmond RC, Woolf BAR, Davies NM, Swanson SA, VanderWeele TJ, et al. Strengthening the Reporting of Observational Studies in Epidemiology using Mendelian Randomisation (STROBE-MR): Explanation and Elaboration. BMJ. 2021;375:n2233.
