# Supplementary material for: Genetic Insights Into Skin Diseases and Depression: Evidence From East Asian Mendelian Randomization Analysis
Source: Alpha Psychiatry. 2025 Oct 24;26(5):47646. doi: 10.31083/AP47646 (PMC12593751; doi:10.31083/AP47646)
Supplement: Supplementary file 1 [file 2757-8038-26-5-47646-s1.zip › Supplementary Table 1.docx]

| **Supplementary Table 1** Summary of SNP details used for analysis | | | | | | |  |  |  |  |  |  |  |  |  |  |  |  |  |  |  |
| --- | --- | --- | --- | --- | --- | --- | --- | --- | --- | --- | --- | --- | --- | --- | --- | --- | --- | --- | --- | --- | --- |
| SNP | chr | pos | Exposure | | Outcome | |  | Exposure | | | |  | Outcome | | | |  |  |  |  |  |
|  |  |  | A1 | A2 | A1 | A2 |  | beta | eaf | se | pval |  | beta | eaf | se | pval |  | steiger_dir | steiger_pval | R2 | F |
| **AD→MDD** | |  |  |  |  |  |  |  |  |  |  |  |  |  |  |  |  |  |  |  |  |
| rs12634229 | 3 | 112376308 | C | T | C | T |  | 0.193 | 0.330 | 0.026 | 1.08E-13 |  | 0.038 | 0.300 | 0.017 | 0.030 |  | TRUE | 7.29E-05 | 1.65% | 2823 |
| rs1295685 | 5 | 131996445 | G | A | G | A |  | -0.226 | 0.694 | 0.027 | 2.11E-17 |  | -0.017 | 0.682 | 0.017 | 0.301 |  | TRUE | 3.49E-08 | 2.17% | 3722 |
| rs140780894 | 6 | 32625384 | T | G | T | G |  | -0.196 | 0.372 | 0.028 | 1.35E-12 |  | -0.042 | 0.241 | 0.025 | 0.087 |  | TRUE | 5.63E-05 | 1.80% | 3076 |
| rs201887180 | 2 | 112369492 | A | G | A | G |  | -0.263 | 0.111 | 0.046 | 9.71E-09 |  | 0.014 | 0.139 | 0.030 | 0.633 |  | TRUE | 0.000106 | 1.37% | 2331 |
| rs2155219 | 11 | 76299194 | T | G | T | G |  | 0.146 | 0.418 | 0.025 | 3.59E-09 |  | -0.001 | 0.558 | 0.015 | 0.969 |  | TRUE | 1.74E-05 | 1.04% | 1772 |
| rs2259735 | 20 | 52788314 | C | T | C | T |  | -0.159 | 0.743 | 0.029 | 4.25E-08 |  | -0.002 | 0.680 | 0.017 | 0.917 |  | TRUE | 8.03E-05 | 0.96% | 1629 |
| rs2395038 | 6 | 31460451 | G | T | G | T |  | -0.145 | 0.637 | 0.026 | 1.72E-08 |  | -0.025 | 0.748 | 0.021 | 0.222 |  | TRUE | 0.000967 | 0.97% | 1647 |
| rs3860444 | 2 | 103007623 | G | A | G | A |  | -0.194 | 0.575 | 0.025 | 5.27E-15 |  | -0.013 | 0.514 | 0.015 | 0.401 |  | TRUE | 2.54E-07 | 1.84% | 3143 |
| rs4312054 | 11 | 7977161 | G | T | G | T |  | -0.181 | 0.463 | 0.025 | 2.09E-13 |  | 0.016 | 0.418 | 0.019 | 0.417 |  | TRUE | 1.38E-06 | 1.63% | 2792 |
| rs4320035 | 3 | 33113697 | C | G | C | G |  | -0.160 | 0.313 | 0.027 | 2.80E-09 |  | -0.002 | 0.384 | 0.016 | 0.894 |  | TRUE | 2.04E-05 | 1.10% | 1864 |
| rs438694 | 10 | 64484681 | C | G | C | G |  | 0.201 | 0.194 | 0.031 | 1.19E-10 |  | 0.003 | 0.163 | 0.023 | 0.898 |  | TRUE | 3.67E-06 | 1.26% | 2141 |
| rs56036302 | 6 | 32272156 | T | A | T | A |  | -0.258 | 0.201 | 0.031 | 1.04E-16 |  | -0.004 | 0.278 | 0.023 | 0.851 |  | TRUE | 2.66E-09 | 2.14% | 3671 |
| rs6010620 | 20 | 62309839 | G | A | G | A |  | 0.172 | 0.336 | 0.028 | 7.34E-10 |  | -0.009 | 0.284 | 0.017 | 0.607 |  | TRUE | 3.20E-05 | 1.32% | 2243 |
| rs6723629 | 2 | 71067853 | C | A | C | A |  | -0.162 | 0.256 | 0.028 | 1.02E-08 |  | 0.008 | 0.263 | 0.018 | 0.652 |  | TRUE | 0.000101 | 1.00% | 1701 |
| rs67494727 | 6 | 31318041 | C | T | C | T |  | -0.248 | 0.183 | 0.032 | 1.18E-14 |  | 0.001 | 0.203 | 0.025 | 0.981 |  | TRUE | 1.74E-08 | 1.84% | 3158 |
| rs6780220 | 3 | 33087200 | C | A | C | A |  | 0.167 | 0.531 | 0.025 | 2.94E-11 |  | 0.013 | 0.550 | 0.015 | 0.394 |  | TRUE | 1.79E-05 | 1.39% | 2367 |
| **Urticaria→MDD** | |  |  |  |  |  |  |  |  |  |  |  |  |  |  |  |  |  |  |  |  |
| rs11030639 | 11 | 4039056 | G | A | G | A |  | 0.096 | 0.306 | 0.016 | 2.74E-09 |  | 0.032 | 0.351 | 0.016 | 0.047 |  | TRUE | 0.002937 | 0.39% | 676 |
| rs143547788 | 16 | 1280087 | C | G | C | G |  | 0.133 | 0.887 | 0.025 | 1.43E-07 |  | 0.008 | 0.833 | 0.027 | 0.782 |  | TRUE | 0.000269 | 0.36% | 614 |
| rs375132703 | 16 | 28656244 | C | G | C | G |  | -0.110 | 0.139 | 0.024 | 3.51E-06 |  | -0.057 | 0.112 | 0.033 | 0.084 |  | TRUE | 0.02808 | 0.29% | 502 |
| rs55908723 | 10 | 50384547 | T | C | T | C |  | -0.085 | 0.658 | 0.016 | 1.13E-07 |  | 0.002 | 0.634 | 0.017 | 0.903 |  | TRUE | 0.000156 | 0.33% | 563 |
| rs7309185 | 12 | 104512832 | A | G | A | G |  | -0.079 | 0.470 | 0.015 | 1.22E-07 |  | -0.019 | 0.482 | 0.016 | 0.237 |  | TRUE | 0.002348 | 0.31% | 538 |
| **Vitiligo→MDD** | |  |  |  |  |  |  |  |  |  |  |  |  |  |  |  |  |  |  |  |  |
| rs1048469 | 9 | 117009115 | A | G | A | G |  | -0.199 | 0.452 | 0.034 | 4.834E-09 |  | 0.008 | 0.321 | 0.017 | 0.637 |  | TRUE | 2.27E-08 | 1.97% | 211 |
| rs10769263 | 11 | 47417183 | C | A | C | A |  | -0.184 | 0.378 | 0.033 | 2.331E-08 |  | 0.011 | 0.307 | 0.017 | 0.509 |  | TRUE | 1.24E-07 | 1.59% | 170 |
| rs111536354 | 6 | 16771953 | G | A | G | A |  | -0.316 | 0.015 | 0.037 | 7.649E-18 |  | 0.011 | 0.380 | 0.020 | 0.565 |  | TRUE | 1.71E-16 | 0.30% | 32 |
| rs112500859 | 4 | 160311666 | G | A | G | A |  | -0.361 | 0.819 | 0.048 | 3.908E-14 |  | -0.005 | 0.454 | 0.023 | 0.839 |  | TRUE | 2.57E-13 | 3.86% | 422 |
| rs11666801 | 19 | 14415129 | A | G | A | G |  | -0.192 | 0.398 | 0.032 | 2.714E-09 |  | -0.021 | 0.339 | 0.017 | 0.205 |  | TRUE | 3.72E-08 | 1.76% | 188 |
| rs12046559 | 1 | 206989067 | T | C | T | C |  | -0.185 | 0.887 | 0.033 | 1.929E-08 |  | 0.017 | 0.473 | 0.016 | 0.280 |  | TRUE | 1.74E-07 | 0.69% | 73 |
| rs12140581 | 1 | 47517960 | C | G | C | G |  | -0.228 | 0.178 | 0.035 | 5.79E-11 |  | 0.023 | 0.376 | 0.020 | 0.262 |  | TRUE | 9.37E-10 | 1.52% | 162 |
| rs12198625 | 6 | 29936795 | C | T | C | T |  | 0.368 | 0.456 | 0.032 | 6.396E-30 |  | 0.002 | 0.351 | 0.021 | 0.914 |  | TRUE | 3.05E-28 | 1.38% | 147 |
| rs12657783 | 5 | 37397221 | A | G | A | G |  | -0.413 | 0.328 | 0.035 | 1.996E-31 |  | -0.009 | 0.437 | 0.016 | 0.600 |  | TRUE | 3.45E-29 | 7.51% | 854 |
| rs12986210 | 19 | 41447427 | G | A | G | A |  | 0.369 | 0.194 | 0.050 | 2.165E-13 |  | 0.020 | 0.318 | 0.023 | 0.386 |  | TRUE | 3.76E-12 | 4.27% | 469 |
| rs13215365 | 6 | 156771003 | G | A | G | A |  | 0.748 | 0.054 | 0.036 | 9.67E-97 |  | -0.003 | 0.280 | 0.018 | 0.856 |  | TRUE | 2.69E-90 | 5.73% | 639 |
| rs13227879 | 7 | 139355465 | T | C | T | C |  | -1.158 | 0.206 | 0.086 | 3.922E-41 |  | -0.057 | 0.097 | 0.037 | 0.119 |  | TRUE | 6.50E-37 | 43.82% | 8194 |
| rs139466 | 22 | 41614259 | A | G | A | G |  | 0.177 | 0.328 | 0.030 | 2.68E-09 |  | 0.003 | 0.447 | 0.016 | 0.874 |  | TRUE | 8.56E-09 | 1.39% | 148 |
| rs150994010 | 18 | 12870401 | T | C | T | C |  | 0.587 | 0.159 | 0.038 | 4.021E-54 |  | -0.024 | 0.344 | 0.020 | 0.238 |  | TRUE | 2.49E-49 | 9.20% | 1064 |
| rs1562787 | 3 | 150087238 | T | C | T | C |  | -0.208 | 0.232 | 0.031 | 1.043E-11 |  | 0.001 | 0.437 | 0.016 | 0.933 |  | TRUE | 4.10E-11 | 1.54% | 164 |
| rs1672814 | 10 | 39012502 | A | G | A | G |  | 5.458 | 0.177 | 0.366 | 3.605E-50 |  | -0.026 | 0.084 | 0.040 | 0.517 |  | TRUE | 1.90E-46 | 1.38% | 147 |
| rs17128310 | 10 | 91620673 | C | G | C | G |  | -0.342 | 0.758 | 0.045 | 3.554E-14 |  | 0.015 | 0.819 | 0.021 | 0.481 |  | TRUE | 5.45E-13 | 4.31% | 473 |
| rs174541 | 11 | 61565908 | C | T | C | T |  | -0.234 | 0.374 | 0.041 | 1.397E-08 |  | 0.018 | 0.439 | 0.016 | 0.270 |  | TRUE | 1.34E-07 | 2.56% | 276 |
| rs17822931 | 16 | 48258198 | C | T | C | T |  | -0.596 | 0.861 | 0.074 | 7.318E-16 |  | 0.024 | 0.125 | 0.036 | 0.506 |  | TRUE | 1.39E-14 | 8.51% | 977 |
| rs201262139 | 3 | 181501884 | C | A | C | A |  | -0.198 | 0.873 | 0.035 | 1.914E-08 |  | 0.000 | 0.461 | 0.020 | 0.987 |  | TRUE | 4.57E-08 | 0.87% | 92 |
| rs2189521 | 16 | 27413566 | C | T | C | T |  | -0.333 | 0.378 | 0.037 | 2.318E-19 |  | 0.005 | 0.306 | 0.018 | 0.794 |  | TRUE | 3.55E-18 | 5.21% | 577 |
| rs229533 | 22 | 37587111 | A | C | A | C |  | -0.212 | 0.577 | 0.035 | 1.098E-09 |  | 0.037 | 0.267 | 0.018 | 0.036 |  | TRUE | 4.85E-08 | 2.20% | 236 |
| rs2523555 | 6 | 31331520 | T | C | T | C |  | 0.408 | 0.163 | 0.039 | 6.414E-26 |  | -0.004 | 0.134 | 0.029 | 0.885 |  | TRUE | 1.91E-24 | 4.54% | 499 |
| rs2613118 | 6 | 56728643 | T | G | T | G |  | 0.600 | 0.532 | 0.043 | 4.268E-44 |  | -0.020 | 0.245 | 0.024 | 0.396 |  | TRUE | 1.50E-40 | 17.92% | 2295 |
| rs2643356 | 15 | 101971388 | C | T | C | T |  | 0.636 | 0.648 | 0.034 | 2.185E-79 |  | 0.015 | 0.426 | 0.016 | 0.336 |  | TRUE | 7.19E-73 | 18.45% | 2377 |
| rs3107771 | 3 | 195430625 | A | G | A | G |  | 0.390 | 0.124 | 0.059 | 3.995E-11 |  | -0.024 | 0.181 | 0.030 | 0.416 |  | TRUE | 4.28E-10 | 3.30% | 358 |
| rs350886 | 19 | 4090113 | T | C | T | C |  | -0.453 | 0.350 | 0.080 | 1.493E-08 |  | -0.047 | 0.067 | 0.034 | 0.167 |  | TRUE | 2.00E-07 | 9.33% | 1082 |
| rs377681 | 19 | 54766423 | A | G | A | G |  | -0.347 | 0.844 | 0.049 | 1.651E-12 |  | 0.002 | 0.287 | 0.023 | 0.950 |  | TRUE | 7.01E-12 | 3.17% | 345 |
| rs4638977 | 3 | 108873606 | C | T | C | T |  | -0.347 | 0.162 | 0.062 | 2.638E-08 |  | -0.015 | 0.077 | 0.030 | 0.630 |  | TRUE | 1.11E-07 | 3.27% | 355 |
| rs4710176 | 6 | 167491639 | C | T | C | T |  | 0.224 | 0.408 | 0.030 | 1.219E-13 |  | -0.012 | 0.469 | 0.016 | 0.459 |  | TRUE | 1.82E-12 | 2.42% | 261 |
| rs497058 | 6 | 32956720 | C | T | C | T |  | -0.166 | 0.573 | 0.030 | 2.834E-08 |  | -0.003 | 0.464 | 0.019 | 0.874 |  | TRUE | 7.97E-08 | 1.36% | 144 |
| rs55755941 | 18 | 52904309 | A | G | A | G |  | -0.408 | 0.288 | 0.039 | 6.802E-26 |  | -0.009 | 0.345 | 0.016 | 0.578 |  | TRUE | 5.23E-24 | 6.85% | 772 |
| rs57986961 | 17 | 44947821 | A | G | A | G |  | -0.234 | 0.308 | 0.036 | 8.679E-11 |  | -0.040 | 0.506 | 0.019 | 0.039 |  | TRUE | 4.99E-09 | 2.34% | 251 |
| rs62133290 | 2 | 69070651 | G | A | G | A |  | 0.181 | 0.303 | 0.033 | 3.293E-08 |  | -0.009 | 0.463 | 0.016 | 0.570 |  | TRUE | 1.52E-07 | 1.38% | 147 |
| rs6727763 | 2 | 199371236 | A | G | A | G |  | -0.535 | 0.170 | 0.083 | 9.825E-11 |  | 0.010 | 0.054 | 0.035 | 0.779 |  | TRUE | 4.55E-10 | 8.05% | 920 |
| rs6951312 | 7 | 158020287 | A | C | A | C |  | -0.914 | 0.397 | 0.033 | 1.852E-170 |  | -0.018 | 0.537 | 0.016 | 0.261 |  | TRUE | 4.27E-155 | 39.99% | 7002 |
| rs7045759 | 9 | 138320339 | T | C | T | C |  | -0.292 | 0.678 | 0.050 | 4.166E-09 |  | 0.002 | 0.390 | 0.017 | 0.897 |  | TRUE | 1.25E-08 | 3.72% | 406 |
| rs720396 | 11 | 113822745 | A | G | A | G |  | 0.206 | 0.544 | 0.035 | 2.359E-09 |  | -0.004 | 0.310 | 0.017 | 0.819 |  | TRUE | 8.39E-09 | 2.11% | 226 |
| rs7255448 | 19 | 33484657 | A | G | A | G |  | -0.535 | 0.255 | 0.042 | 8.498E-38 |  | -0.010 | 0.376 | 0.017 | 0.563 |  | TRUE | 4.67E-35 | 10.87% | 1281 |
| rs72947377 | 2 | 97101590 | T | C | T | C |  | 0.188 | 0.072 | 0.032 | 3.993E-09 |  | 0.008 | 0.382 | 0.016 | 0.621 |  | TRUE | 1.95E-08 | 0.47% | 50 |
| rs73386847 | 7 | 80351991 | A | G | A | G |  | -0.170 | 0.117 | 0.031 | 2.87E-08 |  | -0.007 | 0.411 | 0.016 | 0.678 |  | TRUE | 1.11E-07 | 0.59% | 63 |
| rs7908249 | 10 | 104373300 | A | G | A | G |  | -0.189 | 0.610 | 0.033 | 1.141E-08 |  | -0.019 | 0.322 | 0.017 | 0.258 |  | TRUE | 1.15E-07 | 1.69% | 181 |
| rs825627 | 10 | 13317624 | T | C | T | C |  | -0.568 | 0.509 | 0.045 | 6.746E-36 |  | -0.019 | 0.317 | 0.023 | 0.407 |  | TRUE | 5.40E-33 | 16.13% | 2021 |
| rs9269109 | 6 | 32443266 | T | C | T | C |  | -0.458 | 0.364 | 0.031 | 4.117E-50 |  | 0.025 | 0.446 | 0.019 | 0.197 |  | TRUE | 1.72E-45 | 9.72% | 1131 |
| rs9274945 | 6 | 32641796 | C | T | C | T |  | 0.293 | 0.755 | 0.037 | 1.736E-15 |  | -0.048 | 0.260 | 0.024 | 0.048 |  | TRUE | 2.96E-13 | 1.38% | 147 |
| rs981624 | 6 | 159451328 | A | G | A | G |  | 1.134 | 0.100 | 0.037 | 1E-200 |  | 0.019 | 0.406 | 0.016 | 0.219 |  | TRUE | 4.62E-183 | 23.17% | 3169 |
| **SLE→MDD** | |  |  |  |  |  |  |  |  |  |  |  |  |  |  |  |  |  |  |  |  |
| rs3129758 | 6 | 32584625 | A | G | A | G |  | 0.322 | 0.365 | 0.032 | 2E-23 |  | -0.0002 | 0.303 | 0.021 | 0.991 |  | TRUE | 1.31E-19 | 4.81% | 2062 |
| **Psoriasis→MDD** | |  |  |  |  |  |  |  |  |  |  |  |  |  |  |  |  |  |  |  |  |
| rs10853180 | 17 | 26117407 | A | G | A | G |  | 1.202 | 0.636 | 0.033 | 3.31E-08 |  | 0.002 | 0.694 | 0.017 | 0.930 |  | TRUE | 1.10E-208 | 66.89% | 140811 |
| rs12629982 | 3 | 189663227 | A | G | A | G |  | 0.807 | 0.722 | 0.037 | 5.3E-09 |  | -0.012 | 0.758 | 0.019 | 0.526 |  | TRUE | 1.21E-76 | 26.16% | 24691 |
| rs150995694 | 6 | 31352397 | C | T | C | T |  | 1.928 | 0.020 | 0.070 | 6.86E-21 |  | 0.051 | 0.971 | 0.048 | 0.290 |  | TRUE | 1.91E-117 | 14.57% | 11890 |
| rs3130455 | 6 | 31125978 | A | T | A | T |  | 2.557 | 0.812 | 0.072 | 4.61E-39 |  | -0.008 | 0.920 | 0.036 | 0.828 |  | TRUE | 2.38E-202 | 199.64% | -139625 |
| rs4655524 | 1 | 67629469 | G | A | G | A |  | 1.192 | 0.687 | 0.031 | 1.48E-08 |  | -0.001 | 0.603 | 0.016 | 0.974 |  | TRUE | 3.37E-237 | 61.07% | 109302 |
| rs610984 | 11 | 110003385 | T | C | T | C |  | 1.229 | 0.557 | 0.035 | 3.43E-09 |  | 0.031 | 0.293 | 0.017 | 0.065 |  | TRUE | 1.63E-187 | 74.50% | 203643 |
| rs6873335 | 5 | 158756791 | C | T | C | T |  | 0.775 | 0.783 | 0.031 | 2.27E-16 |  | 0.011 | 0.562 | 0.016 | 0.480 |  | TRUE | 1.53E-97 | 20.38% | 17832 |
| rs72500815 | 6 | 26411596 | G | C | G | C |  | 0.665 | 0.010 | 0.068 | 1.59E-09 |  | -0.012 | 0.124 | 0.044 | 0.784 |  | TRUE | 1.25E-16 | 0.87% | 615 |
| rs76248302 | 2 | 163090855 | G | A | G | A |  | 0.706 | 0.030 | 0.054 | 1.7E-10 |  | 0.038 | 0.865 | 0.025 | 0.137 |  | TRUE | 3.78E-25 | 2.90% | 2084 |
| rs9270527 | 6 | 32559600 | G | A | G | A |  | 1.258 | 0.470 | 0.030 | 4.56E-14 |  | -0.014 | 0.567 | 0.020 | 0.493 |  | TRUE | 3.61E-268 | 78.84% | 259636 |
| rs9368611 | 6 | 29739817 | A | C | A | C |  | 1.877 | 0.023 | 0.040 | 1.88E-55 |  | -0.035 | 0.877 | 0.033 | 0.292 |  | TRUE | 0 | 15.80% | 13074 |
| **Acne→MDD** | |  |  |  |  |  |  |  |  |  |  |  |  |  |  |  |  |  |  |  |  |
| rs747650 | 11 | 47176005 | A | G | A | G |  | 0.215 | 0.395 | 0.037 | 4.41E-09 |  | 0.003 | 0.688 | 0.017 | 0.867 |  | TRUE | 6.53E-09 | 2.21% | 47 |
| rs7531806 | 1 | 169651044 | G | A | G | A |  | 0.199 | 0.435 | 0.035 | 1.20E-08 |  | 0.022 | 0.566 | 0.015 | 0.162 |  | TRUE | 3.59E-08 | 1.94% | 41 |
